# Clinical outcomes of bone transport using rail fixator in the treatment of femoral nonunion or bone defect caused by infection

**DOI:** 10.3389/fsurg.2022.970765

**Published:** 2023-01-09

**Authors:** Ainizier Yalikun, Peng Ren, Maimaiaili Yushan, Aihemaitijiang Yusufu

**Affiliations:** Department of Microrepair and Reconstructive Surgery, The First Affiliated Hospital of Xinjiang Medical University, Urumqi, China

**Keywords:** femur, ilizarov, infected nonunion, rail fixator system, systematic review

## Abstract

**Purpose:**

The rail fixator can improve the treatment outcome and provide good stability in patients with femoral bone transport. The purpose of this study is to investigate the clinical outcomes of bone transport using the Ilizarov technique by rail fixator in the treatment of femoral nonunion or bone defects caused by infection.

**Methods:**

Clinical feature and treatment outcomes of 32 consecutive adult patients with femoral nonunion or bone defect caused by infection from January 2012 to January 2019 at a minimum of 2 years of follow-ups were retrospectively analyzed. Data were collected on participants' demographic details. All difficulties related to bone transport were documented according to Paley's classification. The clinical outcomes were evaluated using ASAMI criteria at the last clinical visit.

**Results:**

All 32 patients with an average follow-up of 33.5 months. There were 17 problems, 21 obstacles, and 8 complications, and the complication rate per patient was 1.4. The main complications were pin-site infection (53.1%), axial deviation (21.9%), joint stiffness (18.8%), the delayed union of the docking site (18.8%), soft tissue incarceration(15.6%), delayed consolidation(6.3%), malunion(6.3%), and refracture (3.1%). All the patients achieved bone union, and no recurrence of infection was observed. The excellent and good rates of ASAMI bone and functional results were 87.5% and 81.3%, respectively.

**Conclusion:**

Bone transport using the Ilizarov technique is an effective method for the treatment of femoral nonunion or bone defect caused by infection, and rail fixators have obtained satisfactory results in terms of bone and functional results.

## Introduction

Femoral nonunion or bone defects caused by infection are often secondary to high-energy trauma associated with acute bone loss or complete removal of infected and sclerotic bone (1–3). The treatment goals include eradication of infection, bone healing, restoration of function, limb alignment, and equal limb length (4). At present, the critical first step in treatment is to eliminate infection and remove all infected bone and soft tissues (5–7). However, this usually leads to limb shortening (8), bone and soft tissue defects (9), and lower limb deformity (7). Several methods have been applied successfully in the treatment of femoral nonunion or bone defect caused by infection, including bone grafting(vascularized fibula graft, iliac bone graft) (10), free tissue transfer, and antibiotic cement (11), Masquelet technique (12), but all techniques possessed its limitations, such as donor site morbidity, stress fracture, and restriction of the size of bone defects. At present, the Ilizarov technique has become the preferred method to solve this series of problems at the same time (13–15).

Due to the rich muscular tissue coverage of the femur compared with the tibia, complications such as long-term pain and knee stiffness are more likely to occur with bone transport with the Ilizarov ring external fixator, which seriously affects the patient's compliance and mental status. However, rail fixators can improve the treatment outcome and provide good stability in patients with femoral bone transport and there are relatively few reports describing its efficacy in the treatment of femoral nonunion or bone defect caused by infection (16,17). In addition, the disadvantages of a ring fixator are that it may be cumbersome, heavy, complicated both for surgeon and patient and may not be well tolerated by the patient when applied for a long period of time (13, 18). In contrast, a monolateral rail fixator has been shown to be less cumbersome for the patient, easier to apply and consequently more surgeon and patient-friendly (19).

Therefore, after the institutional review board approved, we retrospectively analyzed the rail fixators for the treatment of femoral nonunion or bone defect caused by infection, and shared our experience on its efficacy and complications; additionally, we conducted a systematic review of the treatment of femoral nonunion or bone defect caused by infection and obtained a comprehensive evaluation of the effectiveness of Ilizarov technique in the treatment of femoral nonunion or bone defect caused by infection.

## Patients and methods

Inclusion criteria: (1) aged 18–65 years (2) patients with femoral nonunion or bone defect caused by infection treated with rail fixator (3) follow-up time ≥24 months after the removal of external fixator with good compliance. Exclusion criteria: (1) patient with an adjacent joint infection. (2) patients with neurological diseases, cardiovascular and cerebrovascular diseases, and psychological disorders affecting the prognosis of surgery (3) nonunion or defect caused by severe vascular origin disease, primary or secondary tumor, congenital bone disease, or metabolic bone disease. (4) patients with poor compliance or loss of follow-up. A total of 35 patients with femoral nonunion or bone defects caused by infection were treated with a rail fixator in our institution from January 2012 to January 2019. Three patients could not be followed up (could not be contacted or were unwilling to participate in the follow-up). Therefore, a total of 32 patients were included in this study, and all of them signed the relevant informed consent. The study was approved by the Ethics Committee of our institution and conducted in accordance with the ethical principles in the Declaration of Helsinki.

**Figure 1:**
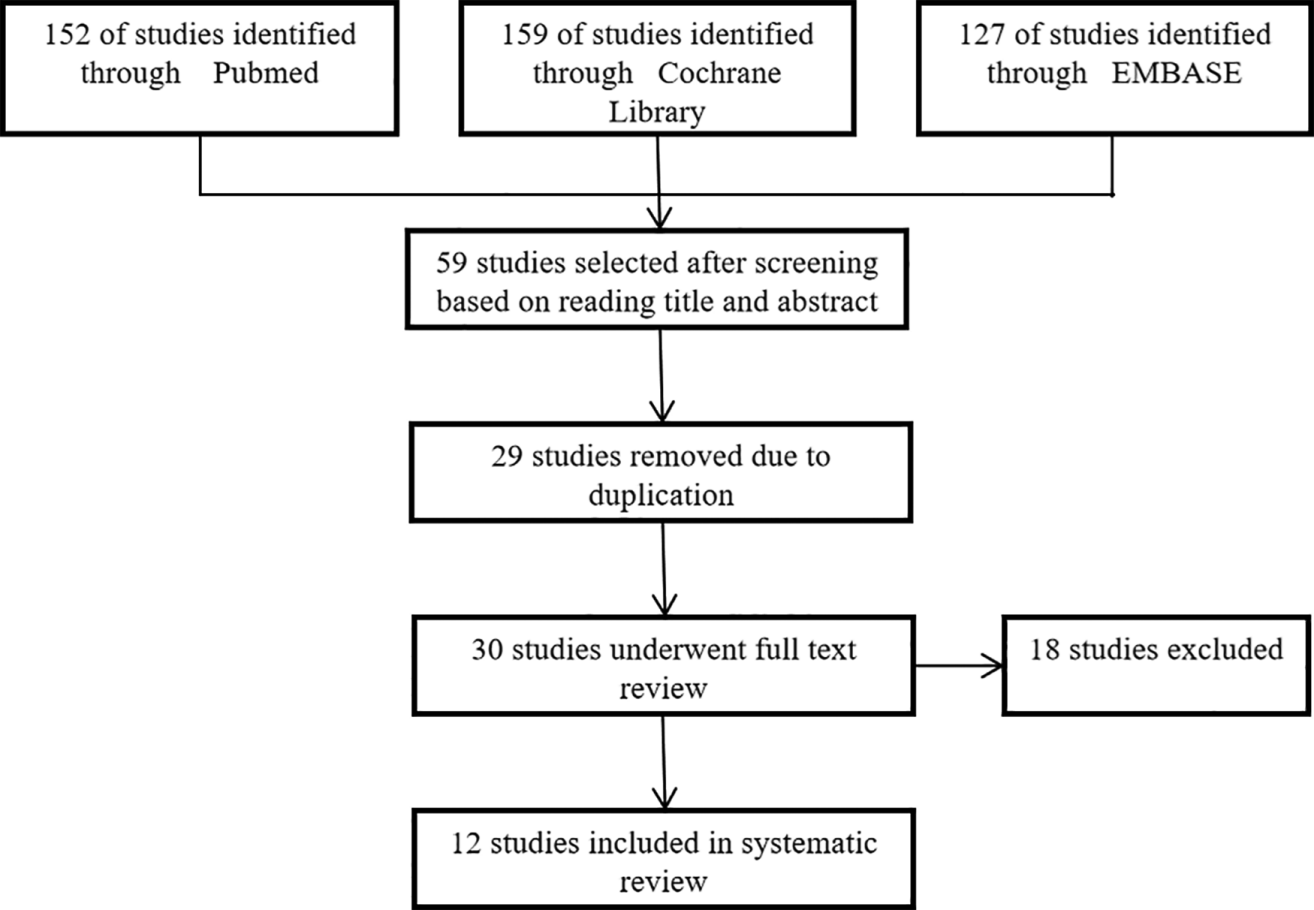
PRISMA flow diagram.

To further demonstrate the effectiveness of the Ilizarov method and provide a basis for the clinical treatment of femoral-infected nonunion, we conducted a systematic review of infected nonunion of the femur treated by the Ilizarov method. We searched the literature from Cochrane Library, EMBASE, PubMed, and other relevant English orthopedic journals between January 2000 and January 2020, and initial literature search provided 438 relevant records, and finally 12 studies and a total of 338 patients were included in the systematic review (20–31). We recorded mean age, mean bone defect length, mean follow-up time, external fixation time (EFT), external fixation index(EFI), bone union rate, bone results, functional results, complications per patient, and occurrence of related complications, and performed statistical analysis using weighted means based on the sample size in each study by SPSS 25.0. The PRISMA is shown in Figure 1.

**Figure 2:**
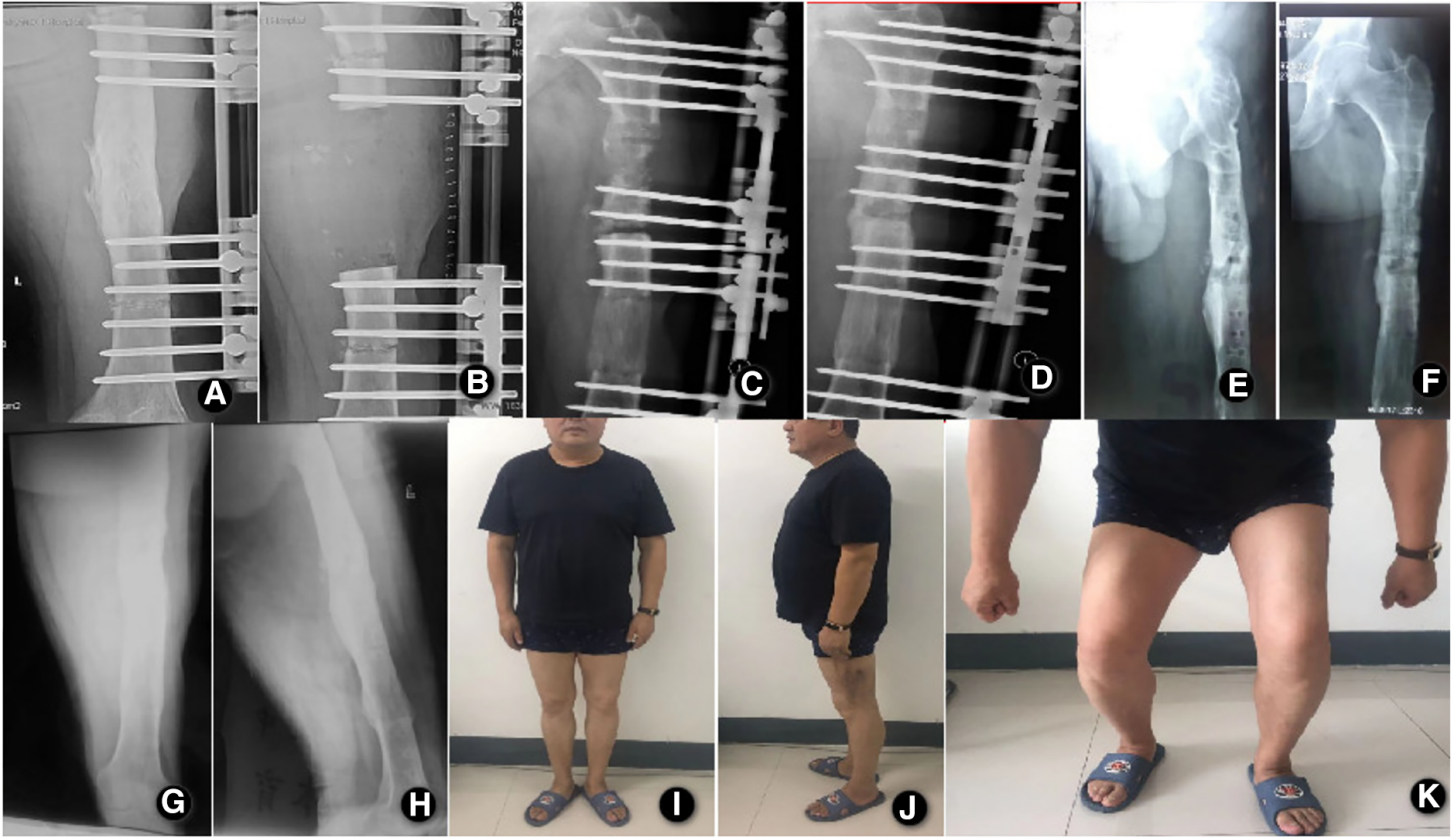
A 42-year-old male patient with a left femur fracture caused by a traffic accident and post-traumatic osteomyelitis after external fixation. (**A**) Anteroposterior x-ray showed osteomyelitis of the middle femur; (**B**) Anteroposterior x-ray films on the day after radical debridement and installation of a rail fixator system showed a bone defect in the length of 10.2 cm; (**C,D**). Anteroposterior and lateral x-ray films 27 days after the operation, showed bone contact was reached; (**E,F**). Anteroposterior and lateral x-ray films at 13.6 months after the operation, showed good bony union and good regenerate consolidation; (**G,H**). Anteroposterior and lateral x-ray films show the good bony union and good regenerate consolidation at 12 months after the removal of external fixation. (**I,J,K**). Functional recovery at 12 months after the removal of external fixation.

**Figure 3:**
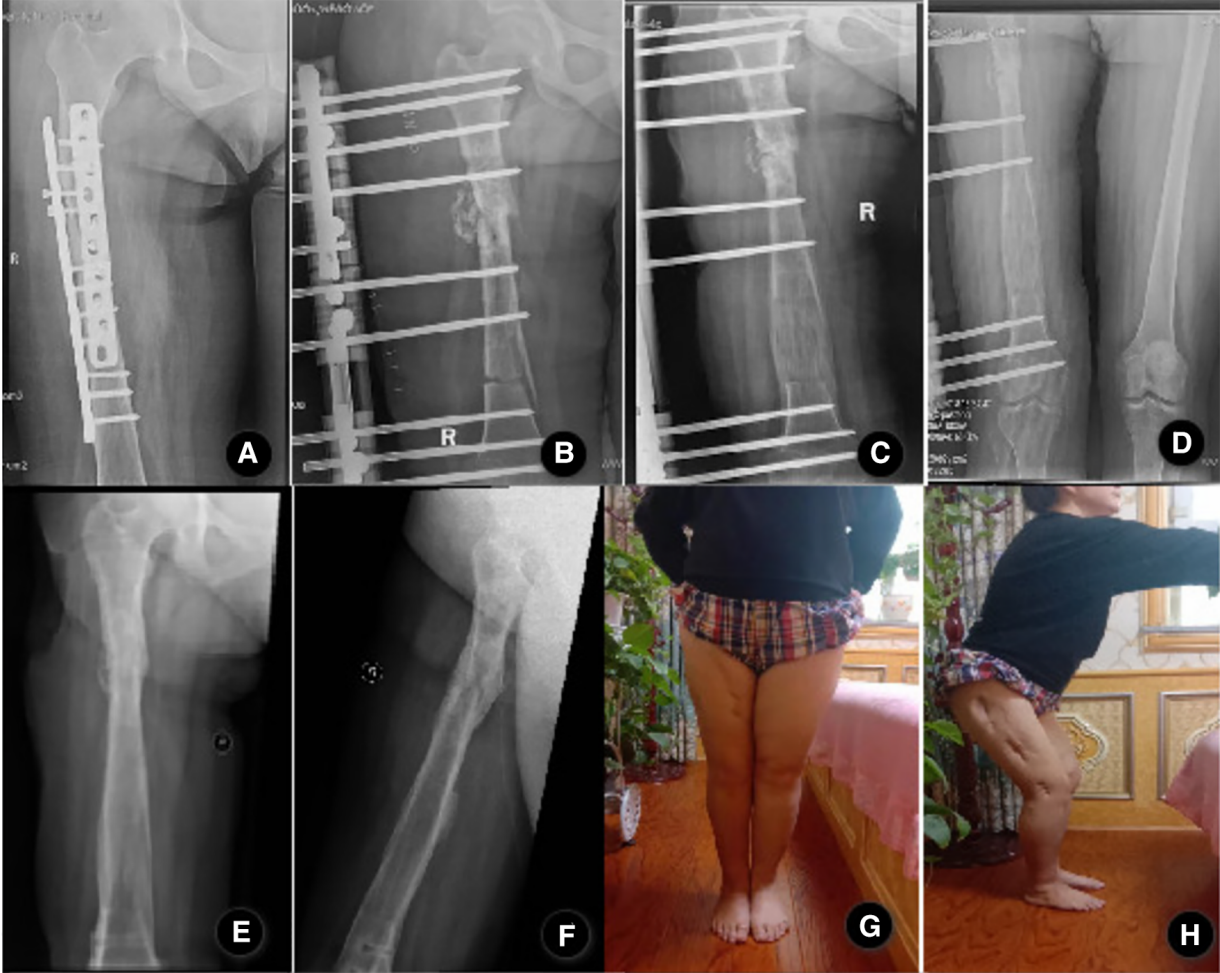
A 39-year-old female patient with a right femur fracture caused by a fall accident and post-traumatic osteomyelitis after plate internal fixation. (**A**) Anteroposterior x-ray showed osteomyelitis of the femur; (**B**) Anteroposterior x-ray films on the day after radical debridement and installation of a rail fixator system, the patient underwent the acute shortening and re-lengthening with the Ilizarov technique after resection of the infected zone, right lower limb was 7.9 cm shorter than the left after surgery; (**C**) Anteroposterior x-ray films at 98 days after the operation, bone healing was observed on both sides of acute shortening and compression. (**D**) Anteroposterior x-ray films at 176 days after the operation, both lower limbs equal in length without shortening. (**E,F**). Anteroposterior and lateral x-ray films showed good bony union and good regenerate consolidation at 12 months after the removal of external fixation;(G.H)The patient showed a good function of flexion and extension of the knee joint;.

There were 29 males and 3 females, with a mean age of 38.2 years (ranging from 19 to 58 years). The mechanism of initial injury included a car accident in 18 cases, fall injury in 7 cases, heavy object injury in 5 cases, and crush injury in 2 cases. The index surgery includes plate fixation (15 patients), intramedullary nail fixation (9 patients), and external fixator fixation (8 patients). After radical debridement, there were 29 cases of bone transport and 3 cases of acute shortening and re-lengthening in our study. Among all patients, there were 23 cases of infected nonunion and 9 cases of chronic osteomyelitis. The average time from initial injury to bone transport surgery was 12.4 months (ranged 9–29 months). The average number of previous operations was 2.4 (ranged 1–8). The average length of the bone defect after radical debridement was 6.8 cm (range: 3.2–12.8 cm). The typical cases are shown in Figures 2, 3.

### Preoperative preparation and surgery

Image tests including x-ray and computed tomography of the affected limb were used before the operation to evaluate the extent of infected or dead bone. According to the imaging results, the extent of infected bone and soft tissue was assessed, the surgical approach was designed and the preinstallation of the rail fixator was completed.

Complete removal of hardware, radical debridement of all necrotic and infected bone and soft tissue, and/or implantation of an antibiotic-impregnated cement spacer to improve stability were performed if necessary prior to bone transport. Cortical bleeding, described as the so-called “paprika sign”, was accepted as an indication of vital osseous and ensured that the medullary cavity is recanalized. Tissue specimens from six different areas were taken and sent for bacterial culture and drug susceptibility tests to guide the surgeon for the appropriate postoperative antibiotics. The wound was irrigated by using hydrogen peroxide, mucosal iodine solution, and physiological saline repeatedly. The rail fixator was installed according to the location of bone defect and soft tissue conditions during operation.

### Postoperative management and follow-up

Intravenous antibiotics were administered for at least 6 weeks or until ESR and CRP levels return to normal based on the result of bacterial culture and drug susceptibility test. Passive knee and ankle exercises are started on the second day after surgery to encourage early partial weight-bearing. Bone transport was initiated 7–10 days after surgery. The fragment was gradually transported to the tibial gap in three to four steps a day, 0.25 mm each, and the compression between docked ends is kept on at the rate of 0.5 mm per day for 5 days in order to get full contact. During the distraction period, the changes in blood supply and skin sensation of the limbs were closely observed. if there was obvious pain around the knee joint or numbness of the toe, the bone transport should be stopped immediately until the sensation and blood supply of the toe returned to normal.

The patients were followed up in the outpatient clinic every 2 weeks, and physical and radiographic examinations were performed to assess pin track condition, external fixator stability, and adjacent joint range of motion. Adjustment of each segmental bone in the distraction period based on radiographic evaluation and patient endurance is essential. Fastening or slowing the rate and rhythm of the individual bone segment based on the regular radiographic evaluation of the distracted gap is likely to apply the “accordion technique” during the distraction period in terms of better regenerating bone formation. The axial deviation and regeneration problems during the distraction period can be well prevented by the above method. When the bridging callus appeared radiographically and limb length equalization was achieved, the frame was dynamized in order to assess the mechanical stability of the regenerated bone and then removed as a daycare procedure. At the time of removal of the external fixator, the leg was protected in a long-leg cast or brace for 4 to 6 weeks with the patient using only partial weight-bearing. Distraction osteogenesis (DOG) time, external fixation time (EFT), external fixation index (EFI), and complications during treatment were recorded, and complications were classified with the Paley classification (14). Bone results and functional results were evaluated according to the Association for the Study and Application of the Method of Ilizarov (ASAMI) classification at follow-up (32, 33).

## Results

All patients were followed up for an average of 33.5 months (range: 24–81 months) after removal of the external fixator, and all patients achieved bone healing without recurrence of infection or new infection. The mean distraction osteogenesis (DOG) time was 69.8 days (range: 35–143 days), the mean external fixation time (EFT) was 12.1 months (range: 6.5–21 months), and the mean external fixation index (EFI) was 1.7 months/cm (range: 1.4–1.9 months/cm). Intraoperative bacterial culture results were taken, including 15 cases (46.9%) of Staphylococcus aureus (including methicillin-resistant Staphylococcus aureus MRSA), 5 cases (18.8%) of Pseudomonas aeruginosa, 4 cases (12.5%) of escherichia coli, 3 cases (9.4%) of Klebsiella pneumonia, and 1 case (3.1%) of Acinetobacter and Proteus, respectively. The remaining 3 patients had culture results suggestive of more than one organism. Details of bacterial species growth in culture are listed in Table 1.

**Table 1:** Proportion of bacterial species growth in culture .

Species	Percent of culture
Staphylococcus aureus (including MRSA)	15 (46.9%)
Pseudomonas aeruginosa	5 (18.8%)
Escherichia coli	4 (12.5%)
Klebsiella	3 (9.4%)
Acinetobacter	1 (3.9%)
Proteus	1 (3.9%)
The result indicates more than one bacterium	3 (9.4%)

Complications were classified according to the Paley classification (34), and a total of 17 problems, 21 obstacles, and 8 complications occurred, and details are shown in Table 2. No patients suffered joint dislocation, neurovascular complications, or compartment syndrome. The most common complication was pin track infection in 17 cases, which was treated by daily pin track care and oral sensitive antibiotics, of which 5 cases had deep pin track infection or pin-wires loosening which was healed after replacement of pins or intravenous antibiotics. There were 7 patients who suffered axial deviations, which were corrected by surgical adjustment and enhanced fixation, and 6 patients had knee stiffness, of which 3 patients had knee range of motion less than 20°, which recovered after postoperative intensive physiotherapy or knee release surgery, and all patients had improvement in knee range of motion, and two patients had improvement in knee range of motion from 20° to 60°. Delayed union at the docking site occurred in 6 patients, 3 of them were treated with compression with an external fixator after bone grafting, and the remaining patients achieved union after compression with an external fixator. 5 patients suffered soft tissue incarceration and managed by freshening the bone ends, opening the medullary canal, and resectioning invaginated soft tissue. There were 2 patients who suffered delayed consolidation and were cured by the “accordion technique” treatment, and 2 patients had malunion of less than 7°, which did not affect their function, so no surgical intervention was performed. One patient suffered refracture at the docking site one month after the external fixator was removed, and the fracture healed after the re-installation of external fixation with bone grafting. In this study, the complications per patient were 1.4 times. In terms of bone results, 18 cases were excellent, 10 cases were good, and 4 cases were fair, and regarding the functional results, 13 cases were excellent, 13 cases were good, and 6 cases were fair. The details are listed in Tables 3, 4.

**Table 2:** Details of the complications.

Parameter	Problems	Obstacles	Complications	Total	Rate of parameter
Pin tract infection	12	5	0	17	53.1%
Axial deviation	2	3	2	7	21.9%
Joint stiffness	0	3	3	6	18.8%
delayed union at the docking site	3	3	0	6	18.8%
Soft tissue incarceration	0	5	0	5	15.6%
Delayed consolidation	0	2	0	2	6.3%
Malunion	0	0	2	2	6.3%
Refracture	0	0	1	1	3.1%
Total	17	21	8	46	

**Problem:** A potential expected difficulty that arises during the distraction or fixation period that is fully resolved by the end of the treatment period by nonoperative means. **Obstacle:** A potential expected difficulty that arises during the distraction or fixation period that is fully resolved by the end of the treatment period by operative means. **Complication:** Any local or systemic intraoperative or perioperative complication, a difficulty during distraction or fixation that remains unresolved at the end of the treatment period, and any early or late posttreatment difficulty.

**Table 3:** Evaluation of the bone and functional results.

Grades	excellent	good	fair	poor
Bone results	18	10	4	0
Functional results	13	13	6	0

**Criteria; Bone results**; Excellent: Union, no infection, deformity <7°, limb length discrepancy (LLD)  < 2.5 cm.; Good: Union plus any two of the following: absence of infection, deformity <7°, LLD <2.5 cm.; Fair: Union plus any one of the following: absence of infection, deformity <7°, LLD <2.5 cm.; Poor: Nonunion/refracture/union plus infection plus deformity >7° plus LLD >2.5 cm.

**Functional results;** Excellent: Active, no limp, minimum stiffness (loss of <15°knee extension/<15°ankle dorsiflexion) no reflex sympathetic dystrophy (RSD), insignificant pain.; Good: Active, with one or two of the following: limb, stiffness, RSD, significant pain.; Fair: Active, with three or all of the following: limb, stiffness, RSD, significant pain.; Poor: Inactive (unemployment or inability to return to daily activities because of injury).; Failure: Amputation.

**Table 4:** Details of the outcomes and complications.

Variable	
Follow-up in months	33.5 (24–81)
Duration of DOG in days	69.8 (35–143)
Time to consolidation in months	1.7 (1.4–1.9)
EFI (months/cm)	32
The number of unions	41
The number of complications	
Pin-track infection	17
Axial deviation	9
Joint stiffness	8
Soft tissue incarceration	5
Delayed union at the docking site	7
Delayed consolidation	2
Malunion	2
Refracture	1
The number of additional surgical interventions	
Bone grafting	4
Knee arthrolysis	3
Accordion technique	2
External fixator adjustment	7
Change external fixation pin	5
Resection of invaginated soft-tissue	5

The calculated datas of systematic literatures are as follows: The mean age of all 338 patients was 36.6 ± 4.2 years (20, 21, 22, 24, 25, 27–31), the mean bone defect was 7.1 ± 2.0 cm (20, 21, 22, 25–31), the mean follow-up time was 53.6 ± 20.8 months (20, 21, 24–30), the mean external fixation time was 13.2 ± 5.8 months (21, 24–27, 29, 30), the mean external fixation index was 1.9 ± 1.2 (21, 23, 25, 26, 28–30), the mean complications per patient was 2.2 ± 0.7 (20–31). The incidence of major complications was as follows, the rate of pin track infection was 73.4% (20–31), the rate of knee stiffness was 32.3% (20–22, 24, 26–31), the rate of the delayed union at the docking site was 14.8% (20–27, 30, 31), the rate of axial deviation was 14.2% (20–23, 31), the rate of malunion (20, 22, 24–26, 28, 31) and the rate of delayed consolidation (20–22, 26, 28, 29, 31) was 7.7%, the rate of recurrent fractures were 3.9% (21, 22, 24, 25, 28–31). The good to excellent rate of bone results was 82.8% (range: 61.5%–94.3%) (21–25, 28, 31), the good to excellent rate of functional results was 71.6% (range: 50%–84.6%) (21–25, 28, 31), and only five of all patients were amputated. The details are shown in Table 5.

**Table 5:** Descriptive characteristics of component studies.

Authors	ST	PN	Male/female	MA	MBD	MPSP (per patient)	Bone union rate (%)	Bone results (excellent/good/fair/poor)	Functional results (excellent/good/fair/poor)	Complications Per patient	EFT (months)	EFI (months/cm)	Follow-up (months)
Karim et al (2019)	PS	50	48/ 2	33.6	3.6	2.8	98	17 (34%)/30 (60%)/1 (2%)/2 (4%)	15 (30%)/24 (48%)/8 (16%)/3 (6%)	1.9	nr	nr	nr
Cengiz et al (2019)	RC	32	22/10	40.3	5.7	2.6	100	20 (63%)/9 (28%)/3 (9%)/0 (0%)(paley)	19 (60%)/11 (34%)/2 (6%)/0 (0%)(paley)	1.6	nr	nr	65.2
Zhang et al (2017)	RS	41	31/10	44	10.1	nr	100	30 (73%)/6 (15%)/5 (12%)/0 (0)	21 (51%)/9 (22%)/7 (17%)/4 (10%)	1.2	13	1.3	35
Yin et al (2015)	RS	35	nr	nr	nr	nr	100	22 (63%)/11 (31%)/2 (6%)/0 (0%)	12 (34%)/15 (43%)/8 (23%)/0	1.3	nr	1.5	nr
Mohamed et al (2013)	RS	20	19/1	37.5	nr	2.0	96	10 (50%)/6 (30%)/1 (5%)/3 (15%)	4 (20%)/13 (65%)/3 (15%)/0 (0%)	3.2	8.0	nr	36.7
Wan et al (2012)	RS	13	8/5	32.2	8.9	2.6	100	6 (46%)/2 (15%)/0 (0%)/5 (39%)	7 (54%)/4 (31%)/2 (15%)/0 (0%)	2.0	14.8	4.4	81.4
Arora et al (2012)	RS	15	13/2	18–47	7.9	2.9	100	12 (80%)/3 (20%)/0 (0%)/0 (0%)(Arora)	5 (33%)/8 (54%)/2 (13%)/0 (0%)(Arora)	2.3	7.3	0.9	19.3
Blum et al (2010)	RS	50	41/9	29.9	8.8	3.8	98	nr	nr	2.3	24.5	nr	70.8
Krishnan et al (2019)	RS	20	17/3	38.4	6	4.4	95	13 (65%)/4 (20%)/1 (5%)/1 (5%)	3 (15%)/9 (45%)/3 (15%)/4 (20%)	3.6	nr	1.3	62.8
Saridis et al (2006)	RS	13	10/3	34.5	8.3	3.1	100	8 (62%)/4 (31%)/1 (7%)/0 (0%)(paley)	3 (23%)/4 (31%)/4 (31%)/2 (15%) (paley)	1.7	10.3	1.6	42.4
Mekhail et al (2006)	RS	19	14/5	36.4	5.7	2.5	89.5	nr	2 (11%)/11 (57%)/4 (21%)/2 (11%) (paley)	2.1	13.8	2.4	68.7
Barbarossa et al (2001)	RS	30	6/24	39.4	6.3	4.4	96.7	13 (43%)/9 (30%)/2 (7%)/5 (17%)/1 (3%)	5 (17%)/10 (33%)/8 (27%)/6 (20%)/1 (3%)	2.9	nr	nr	nr

ST, study type; PN, patient number; MA, mean age; MPSP, mean previous surgery per patient; EFT, external fixator time; EFI, external fixator index; nr, not reported; PS, a prospective study; RS, retrospective study; RC, retrospective cohort.

## Discussion

The treatment of femoral nonunion or bone defects caused by infection is a difficult problem for orthopedic surgeons (20). Patients may usually undergo multiple operations due to high-energy trauma associated with bone and soft tissue defects, lower limb deformities, chronic infections, and other problems during the entire treatment course. Traditional surgical methods often cannot effectively solve this series of problems at the same time (35).

The treatment of femoral nonunion or bone defect caused by infection can only determine the next step in limb reconstructive treatment under the premise of radical debridement and control of infection. Commonly used methods include the Masquelet technique (12), autologous bone graft (vascularized fibula graft, iliac bone graft) (10), and Ilizarov bone transport technique (36), all of which have their advantages and limitations. The Masquelet technique avoids the patient from carrying a heavy circular external fixator, but this technique has higher requirements for the integrity of the soft tissue around the bone defect and requires at least 2 surgical treatments (37). This technique is often limited due to limited bone volume in the donor site, and is associated with risks such as recurrence of infection, failure of revascularization, and ossification of the transplanted bone region and cannot correct the limb deformity that appears during treatment (38). Vascularized fibula transplantation requires a high level of microsurgical techniques, the amount of bone supply is limited, and also can cause secondary injury to the donor site. The technique of vascularized fibula grafting may lead to treatment failure due to insufficient revascularization of the grafted bone segment, and there will be a risk of bone strength difference and refracture if the femoral ossification of the grafted bone segment fails (39, 40). Ilizarov circular external fixator has the shortcomings such as heavy device, postoperative persistent pain, complex installation, and long learning curve. Some studies (24, 25) point out that due to a large amount of muscle and soft tissue coverage around the femur, circular external fixator will bring persistent pain to patients, patients cannot tolerate for a long time, and even seriously affect the patient's mental status and compliance, so some researchers suggest (35, 26) that rail fixator is preferred for patients with femoral bone defects, which also enlightens the development of rail fixator. However, bone transport with rail fixator also can manage bone and soft tissue defects, infections, and correct limb deformities due to trauma simultaneously. It can be applied to all types of nonunion with less demanding soft tissue coverage and a higher fracture healing rate. We obtained satisfactory results by using the rail fixator in treatment of femoral nonunion or bone defect caused by infection, with a good to excellent rate of 87.5% for bone results and 81.3% for functional results. All patients achieved bone union, and no recurrence of infection was observed, similar to the previous study (21). In this study, the bone result was better than the functional result, because the functional result mainly depends on the degree of the original damage to the blood vessels, nerves, muscles, bones, and joints, the good bone result does not reflect the good functional result.

The incidence of pin track infection after femoral bone transport ranged from 33.3% to 100% (20–31). Karim et al. (25) reported that the most important complications in study consisted of pin track infection (80%, 40/50), knee stiffness (60%, 30/50), and pin-wires loosening (20%, 10/50), and they believed that complications are intrinsic to the Ilizarov method however their frequency and severity decrease with increasing experience of the surgical team as well as improvement in the physiotherapy back up facility (25). In addition, some scholars emphasized (41, 42) that serious complications often lead to compliance issues on part of the patients. These factors at times lead to deliberate shortening of the treatment and discontinuation before the achievement of the desired leg length (41, 42). Cengiz et al. (20) reported the incidence of pin track infection in their study was 90.6% (29/32), and they emphasized that pin track infections are unavoidable when using an external fixator and can be the cause of deep infections spreading to the intramedullary nail, therefore, shorter treatment time and radical debridement may reduce the incidence of related complications. Bhardwaj et al. (11) proposed that pin track infection and pain were the most common complications in a study comparing Ilizarov ring fixator (IRF)and rail fixator (RF) in infected nonunion of long bones, and the mean duration of external fixators application was 17.0 months in IRF and 11.6 months in RF. He reported that the bone healing time and external fixator time (EFT) in the RF group were significantly less than those in the IRF group, the RF group had fewer complications and the patients felt more comfortable and acceptable. The most common complication in our study was pin track infection, with an incidence of 53.1% (17/32), due to the rich muscular tissue coverage of the femur compared with the tibia, the screw encounters resistance during bone transport and has more extensive friction with the surrounding soft tissues, on the other hand, although we paid great attention on the postoperative pin site care, pin track infection is also related to the patient's bone quality, general nutritional status and immunity.

In a study of infected nonunion of the tibia and femur treated by bone transport, Yin et al. (23) reported the rate of femur axial deviation was 34.3% (12/35). They suggested that this could be caused by overload weight bearing or excessive functional exercise, but can be prevented by appropriate postoperative rehabilitation and regular x-ray examination. Barbarossa reported that after the end of the treatment, axial deviations of >7 remained in 13 (43.33%, 13/30)patients because of the bending due to the insufficient stability of fragments. He achieved the improvement of the axis <7 in five patients by correcting the frames and adding the wires in general or spinal anesthesia (31). The axial deviation is 21.9% (7/32) in our study, which was higher than the average data recorded in the aforementioned systematic review, which may be due to the limitation in the stability of the rail fixator, and the axial deviation of the rail fixator is more likely to occur than that of the circular external fixator during bone transport, and another reason is excessive functional exercise. However, the axial deviation can be effectively avoided by regular and timely follow-up and reasonable exercise.

Zhang et al. (21) reported the clinical outcome of 41 patients with femoral bone defects treated with rail fixator, of which 14 (34.2%) had knee joint stiffness and a range of knee motion declined; according to the differences in the patients' living requirement, joint arthrolysis was performed later for 10 patients. Mohamed et al. (24) showed a 60% (12/20) rate of joint stiffness in their study, nine of the studied 20 patients presented with initial knee joint stiffness and marked muscle wasting before the index surgery. The degree of joint stiffness was accentuated after the index surgery in those patients. One patient was subjected to arthrolysis of the knee joint and a quadricepsplasty and the knee range of motion was improved from 30 to 80°. In this study, 18.8% (6/32) of the patients had knee joint stiffness, lesions in the distal femur, and different degrees of knee joint stiffness when coming to our hospital for medical treatment. 3 of them received arthrosis after bone transport, and 2 of them had a joint range of motion improved from 20° to 60°. We believe that knee joint stiffness is closely related to the severity of the injury, the location of the bone defect, and the patient's regular functional exercise (compliance) after surgery.

In our study, due to the factors of contact surface deviation and poor blood supply, delayed union at the docking site was also a relatively common complication, reaching 18.8% (6/32). Currently, the need for bone grafting at the docking site is controversial. CarloBiz et al. (43) performed bone grafting at the docking site in 72 patients, and the final bone healing was 100%. In our study, only 3 patients were treated with bone grafting followed by a rail fixator, and all patients had a bone union. However, we should pay more attention to patients with delayed consolidation in the distraction area, because this complication is more likely to occur in older patients with larger bone defects and poor soft tissue coverage. In this study, 2 patients (6.3%) had delayed consolidation, aged 55 and 58 years, with bone defect lengths of 10.5 and 11.3 cm, respectively, and they were treated with the “accordion technique” to achieve bone healing; the “accordion technique” was indeed an effective method for delayed consolidation, which could induce intramembranous and endochondral osteogenesis and promote bone healing (44). In this study, the rate of soft tissue incarceration, malunion, and refracture were 15.6%, 6.3%, and 3.1%, respectively, which were similar to the above systematic review. The complications per patient were 1.4, lower than the average data in the above systematic review (2.2), which may be closely related to our individualized surgical plan, radical debridement, timely postoperative follow-up, early detection of complications, and improvement of patient compliance through psychological counseling.

Advantages of the rail fixator (19, 45): (1) The surgical operation is simple, it is easy to avoid important nerves and blood vessels, and it is more convenient to perform multiple adjustment surgeries (2) The external fixator is fixed on the lateral femur, which basically does not affect the sleep quality of patients and causes less psychological burden to patients (3) The rail fixator is light in weight and small in size to facilitate postoperative functional exercise and avoid late joint dysfunction. (4) Convenient for postoperative wound care and have a short clinical learning curve. Limitation of the rail fixator (16, 29, 46): (1) When treating bone defects adjacent to the joint, the stability of the rail fixator is not as good as that of the Ilizarov ring external fixator. (2) Axial deviation is likely to occur during bone transport.

Limitations of this study include the small sample size, retrospective study, and lack of direct comparison with other treatment options; a multicenter trial with a larger sample size or a randomized controlled trial should be performed to overcome the limitations of our study.

## Conclusion

Ilizarov technique is an effective method for the treatment of femoral nonunion or bone defect caused by infection, and the rail fixator has obtained satisfactory results in terms of bone and functional results after treatment. Radical debridement is the cornerstone of successful treatment, timely follow-up and postoperative psychological counseling help to improve patient compliance and minimize the occurrence of related complications.

## Data Availability

The raw data supporting the conclusions of this article will be made available by the authors, without undue reservation.
